# Slipped Capital Femoral Epiphysis in an Adult Patient With Kabuki Syndrome

**DOI:** 10.5435/JAAOSGlobal-D-19-00084

**Published:** 2019-10-14

**Authors:** Joshua N. Speirs, S. Craig Morris, Martin J. Morrison

**Affiliations:** From the Department of Orthopaedic Surgery, Loma Linda University, Loma Linda, CA.

## Abstract

**Introduction:**

Slipped capital femoral epiphysis (SCFE) is a condition which predominantly occurs in adolescents. SCFE is extremely rare in adults, and nearly all previous reported cases have been associated with an endocrine disorder.

**Methods:**

We present a case of a 19-year-old man with Kabuki syndrome who was diagnosed with an unstable SCFE requiring surgical fixation. A literature search on PubMed and Google Scholar was done looking for all published cases of delayed-onset SCFE. All previous reported cases were reviewed to determine the likely cause of the SCFE.

**Results:**

No previous cases of SCFE were described in patients with Kabuki syndrome. Literature review revealed 27 articles describing 32 cases of delayed-onset SCFE. Thirty-one of these cases were associated with endocrine disorders or pituitary tumors. Endocrine disorders associated with delayed-onset SCFE included hypothyroidism, hypogonadism, and panhypopituitarism. Pituitary adenomas and craniopharyngiomas were also associated with delayed-onset SCFE.

**Discussion:**

This is the first reported case of SCFE in a patient with Kabuki syndrome. Kabuki syndrome is a genetic disorder known to cause delayed growth, resulting in delayed physeal closure, placing the patients at risk of SCFE even into adulthood. The literature search revealed that nearly all previously described delayed-onset SCFE cases were associated with endocrine disorders or pituitary tumors. We recommend that all patients diagnosed with delayed-onset SCFE be evaluated for endocrine disorders, pituitary tumors, and/or genetic disorders which can cause delayed skeletal maturation as these disorders can range from severe endocrine disorders to intracranial tumors.

Slipped capital femoral epiphysis (SCFE) is a hip condition which predominantly occurs in adolescents. SCFE is characterized by posteroinferior displacement of the femoral epiphysis on the metaphysis through an open physis.^1^ SCFE is extremely rare in adults. Only a few studies documenting SCFE in individuals older than 18 years, usually associated with hormonal abnormalities or intracranial tumors resulting in delayed osseous maturation, are found.^2^ Our case differs from most previous reported delayed-onset SCFE because the patient had no endocrine disorder but did have Kabuki syndrome, a known genetic disorder causing delayed skeletal maturation.

## Case

A 19-year-old man presented to the emergency department for evaluation of right hip pain for the past 2 weeks with the inability to walk. The patient had no previous difficulty ambulating. Medical history included Kabuki syndrome, obesity, developmental delay, autoimmune hemolytic anemia, idiopathic thrombocytopenic purpura, and double outlet right ventricle requiring previous open heart surgery. The patient was been followed closely within our institution, including evaluation by endocrinology. The patient had no known endocrine disorder. Comprehensive laboratory and genetic studies had been done without any endocrine disorder identified. On physical examination, the patient's right leg was externally rotated. Any movement of the right hip caused severe pain and guarding, limiting the examination. The patient was unable to ambulate, even with crutches.

**Figure 1:**
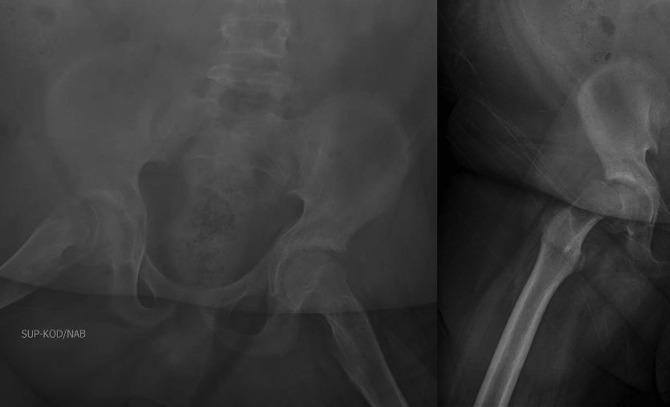
Radiographs at time of presentation—AP pelvis and lateral view of the right hip.

**Figure 2:**
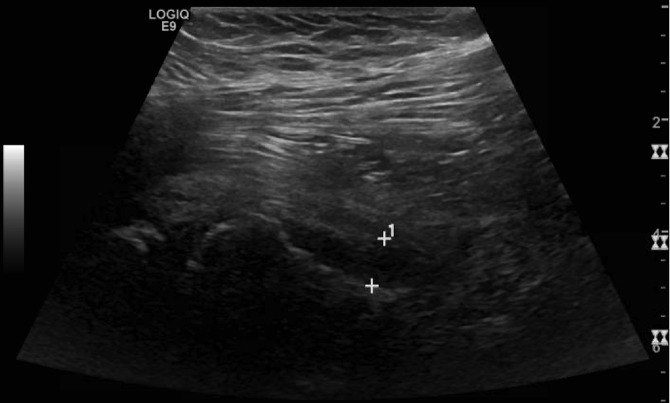
Ultrasonography at time of presentation showing right hip effusion.

**Figure 3:**
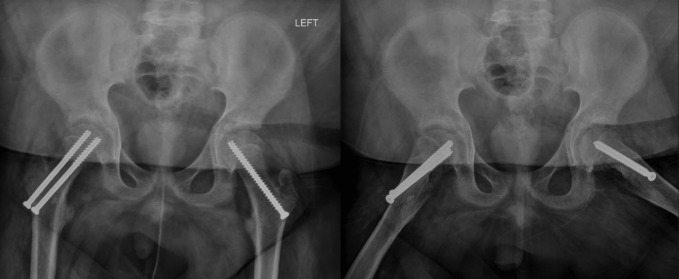
Postop radiographs (AP pelvis and frog-leg lateral).

Radiographs of the pelvis and right hip showed a right SCFE, with epiphyseal-diaphyseal angle (Southwick slip angle) of 75° (Figure 1). An ultrasonography was ordered which revealed the presence of a right hip effusion (Figure 2). These images and examination confirmed the diagnosis of an acute severe unstable SCFE. The patient was taken to the operating room where an anterior Smith-Peterson approach was done and a capsulotomy was made revealing the presence of a hemarthrosis. A reduction was done with gentle pressure on the anterior aspect of the metaphysis as previously described by Parsch et al.^3^ Two cannulated screws were placed across the physis to maintain the reduction. The contralateral hip was also pinned prophylactically given the atypical SCFE (Figure 3).

The patient was made non–weight-bearing on the right hip for 6 weeks while maintaining weight bearing as tolerated on the left side. He began walking independently 8 weeks postoperatively. At last follow-up, 7 months postoperatively, the patient was back to baseline function, and radiographs did not reveal any signs of osteonecrosis of the femoral head. The physes of bilateral proximal femurs were still open.

## Methods

A literature review on PubMed and Google Scholar was done looking for all published cases of delayed-onset SCFE in patients older than 18 years. Key words included in the search were delayed-onset slipped capital femoral epiphysis, adult slipped capital femoral epiphysis, adult SCFE, and delayed-onset SCFE. All identified published cases of delayed-onset SCFE were reviewed to determine the likely cause of the SCFE. Articles were excluded if case details were not provided. In addition, the references of each published case were cross-checked to identify additional cases.

## Results

The literature review did not reveal any previous published cases of SCFE in any patients with Kabuki syndrome. Twenty-seven articles were identified between the years 1953 and 2018 describing 32 cases of delayed-onset SCFE.^2,4,5,6,7,8,9,10,11,12,13,14,15,16,17,18,19,20,21,22,23,24,25,26,27,28,29^ Including the case presented in this article, 31 of the 33 cases (93.9%) of delayed-onset SCFE were associated with an endocrine disorder or pituitary tumor. See Table 1 for the endocrine disorders and pituitary tumors associated with delayed-onset SCFE identified in the literature review. The case presented in this article describes one of the two SCFEs not associated endocrine disorders. The second case was a 47-year-old woman with persistent open physis. Although the exact reason for the patient's skeletal immaturity was unknown, the authors postulated that it may be related to her chronic use of inhaled corticosteroids.^25^ Seven additional cases of delayed-onset SCFE in patients older than 20 years were published by Noguchi et al in 2002 in a study reporting on the epidemiology and demographics of SCFE in Japan, but as the details of these cases are not included in the publication, they were not included in the analysis.^30^

**Table 1:**
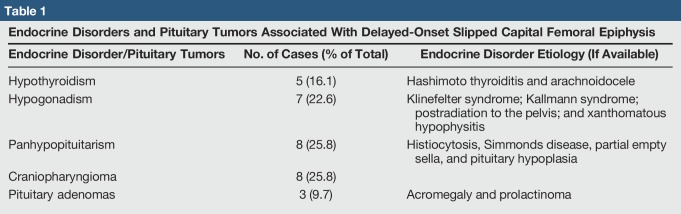
Endocrine Disorders and Pituitary Tumors Associated With Delayed-Onset Slipped Capital Femoral Epiphysis

Endocrine Disorder/Pituitary Tumors	No. of Cases (% of Total)	Endocrine Disorder Etiology (If Available)
Hypothyroidism	5 (16.1)	Hashimoto thyroiditis and arachnoidocele
Hypogonadism	7 (22.6)	Klinefelter syndrome; Kallmann syndrome; postradiation to the pelvis; and xanthomatous hypophysitis
Panhypopituitarism	8 (25.8)	Histiocytosis, Simmonds disease, partial empty sella, and pituitary hypoplasia
Craniopharyngioma	8 (25.8)	
Pituitary adenomas	3 (9.7)	Acromegaly and prolactinoma

## Discussion

Kabuki syndrome is a genetic disorder characterized by multiple abnormalities including distinctive facial features, skeletal abnormalities, delayed growth, and intellectual disability. Kabuki syndrome is most common among patients of Japanese descent and is secondary to a nonfunction protein involved in chromatin remodeling.^31^ Our patient was treated for an unstable SCFE and with prophylactic fixation of the contralateral side. Treatment for SCFE regardless of age usually includes in situ fixation with possible gentle reduction.^1^ Prophylactic pinning of the contralateral hip was done after a discuss with the family because of the patient's continued skeletally immaturity and limited ability of the patient to communicate pain because of his intellectual delay. Kocker et al recommend the decision for contralateral hip pinning be made with the family after consideration of the patient's age, sex, endocrine status, and other risk factors.^32^

This is the first reported case of SCFE in a patient with Kabuki syndrome. The literature search revealed that ∼94% of all previously described delayed-onset SCFE were found to have either an endocrine disorder or pituitary tumor resulting in delayed skeletal maturation. We recommend that all patients diagnosed with delayed-onset SCFE be evaluated for endocrine, pituitary tumors or genetic disorders which could cause delayed skeletal maturation. In addition, when treating patients with known endocrine abnormalities or syndromes resulting in delayed osseous maturation, reports of hip or knee pain should not be dismissed because these patients are at risk of SCFE, regardless of age.
